# Thermal Properties and Dynamic Characteristics of Electrospun Polylactide/Natural Rubber Fibers during Disintegration in Soil

**DOI:** 10.3390/polym14051058

**Published:** 2022-03-07

**Authors:** Yulia V. Tertyshnaya, Svetlana G. Karpova, Maria V. Podzorova, Anatoliy V. Khvatov, Maksim N. Moskovskiy

**Affiliations:** 1 Department of Biological and Chemical Physics of Polymers, Emanuel Institute of Biochemical Physics, Russian Academy of Sciences, 4 Kosygina Str., 119334 Moscow, Russia; karpova@sky.chph.ras.ru (S.G.K.); yersinia@bk.ru (M.V.P.); kvatov@mail.ru (A.V.K.); 2 Perspective Composite Materials and Technologies Laboratory, Plekhanov Russian University of Economics, 36 Stremyanniy, 117997 Moscow, Russia; 3 Federal Scientific Agroengineering Center VIM, 1st Institutskiy Proezd, 5, 109428 Moscow, Russia; maxmoskovsky74@yandex.ru

**Keywords:** polylactide, biobased fiber, natural rubber, electrospinning, polymer substrate, thermal properties, degradation in soil

## Abstract

In this work, PLA/NR electrospun fibers were used as substrates for growing basil. Thermal characteristics of initial samples and after 60 and 220 days of degradation were determined using differential scanning calorimetry. In the process of disintegration, the melting and glass transition temperatures in PLA/NR composites decreased, and in PLA fibers these values increased slightly. TGA analysis in an argon environment confirmed the effect of NR on the thermal degradation of PLA/NR fibers. After exposure to the soil for 220 days, the beginning of degradation shifted to the low-temperature region. The dynamic characteristics of the fibers were determined by the EPR method. A decrease in the correlation time of the probe-radical in comparison with the initial samples was shown. FTIR spectroscopy was used to analyze the chemical structure before and after degradation in soil. In PLA/NR fibrous substrates, there was a decrease in the intensity of the bands corresponding to the PLA matrix and the appearance of N-H C-N groups due to biodegradation by soil microorganisms.

## 1. Introduction

Growing concerns about environmental protection and partial refusal of petroleum-based polymers led to the invention of bio-based polymers [1,2]. Eco-friendly polymers such as poly-3-hydroxybutyrate, polycaprolactone and polylactide are actively being introduced into today’s world [3,4,5].

Fibrous materials based on PLA can serve as covering materials for growing new experimental varieties in breeding and seed production or serve as substrates for growing various crops [6]. Modern trends focused on eco-friendly technologies are leading to increasing use of biodegradable materials in agricultural production. This is especially true for crop production, which uses covering and mulching polymer materials, a significant part of which enters the soil directly during their use, bypassing the stages of collection, sorting and processing.

Polylactide (PLA) is a linear aliphatic polyester obtained in two stages. First, the monomer lactic acid is released during the fermentation of waste vegetable raw materials. At the next stage, polylactide is obtained from lactic acid by polymerization [7,8,9]. PLA is a biodegradable and biocompatible thermoplastic; it is produced on a large scale and used for various applications in different industries, such as medicine, packaging and agriculture [10,11,12]. However, the high brittleness, rigidity and gas permeability of PLA force it to be modified. Natural rubber, which is an elastomer derived from latex and is obtained from the rubber tree juice, is used for PLA modification. It is known that rubber particles behave as stress concentrators, absorbing the fracture energy of brittle PLA and this results in a material with improved toughness [13,14]. In addition, it increases the elasticity of composites and the macromolecular mobility of the thermoplastics–elastomer system [15].

In this work non-woven PLA/NR fibers with different natural rubber content are obtained by electrospinning. Electrospinning is a complex process that involves the hydrodynamics of weakly conducting Newtonian liquids and phase transformations, that is, the evaporation of the solvent and formation of the polymer fiber. It is used to produce ultrathin fibers for many applications: nanosensors, biomedical, tissue engineering, filtration systems and others [16,17,18]. Electrospun fibers have unique properties, such as having a high surface area to volume ratio and uniform morphology [19]. It is known that ultrathin fibers enhance protein absorption, cell growth and adhesion [20].

Polylactide is a relevant polymer for all applications because it is biodegradable and easily electrospinnable. The electrospinning process and properties of non-woven fibers from PLA and PLA composites are described in [21,22,23,24]. Patra and coauthors studied the effect of manufacturing parameters on the morphology and diameter of PLA nanofibers [21]. The authors of the work [22] evaluated the possibility of contamination with molds of PLA/PHB fibrous materials. The high aggressiveness of micromycetes *Aspergillus niger* and *Chaetomium globosum* was shown. Thermal degradation of PLA was also studied. PLA is sensitive to thermal degradation above its melting point. Therefore, the study of its thermal stability is important for its processing and application in various industries. The authors [25,26,27] investigated the thermal degradation of PLA and PLA composites. For example, the work [25] shows the differences in the course of the destruction of the PLA/starch composite in an inert medium and an oxygen atmosphere. Blanco [26] investigated the isothermal process of PLA degradation.

Despite the abundance of works on thermochemistry and the structure of PLA and PLA composites, no thermal degradation and dynamic properties of PLA/NR fibers after degradation in soil were reported. In this work, the influence of soil exposure on the morphology, thermal and structural-dynamic characteristics of PLA/NR fibrous materials is considered.

## 2. Materials and Methods

### 2.1. Sample Preparation

Thermoplastic PLA, poly(lactic acid), 4032D (with about 2% of D-lactide) with ρ = 1.24 g/cm^3^ and molecular weight (M_w_) of 1.7 × 10^5^ g/mol was procured from Nature Works (Minnetonka, MN, USA). Elastomer natural rubber (NR), SVR-3L with poly(*cis*-1,4-isoprene) content: 91–96, wt.%, Mooney viscosity 50 ± 5 (100 °C) and volatiles 0.8 wt.% was kindly supplied by the Vietnam Rubber Group (Ho Chi Minh City, Vietnam). Polymers were used without any purification.

Fiber electrospinning: the polymer solutions were prepared by dissolving neat PLA and PLA/NR in the right ratio in 100 mL of chloroform. The polymer mixtures were heated at 60 °C for about 4–5 min. The obtained solution was placed in a syringe with an inner needle diameter of 0.7 mm, set up vertically. The electrospinning experiments were performed at a temperature of 20 ± 2 °C. The sample weight was 9 g per 100 mL, the ratio of the components (PLA:NR, wt.%) was 100:0, 95:5, 90:10 and 85:15 T and the consumption of the solution was in the range (9–11) × 10^−5^ g/s. The electrospinning process was run for 5–5.5 h for each sample with a voltage of 17.5–19 kV and 17 ± 1 cm between the needle tip and the collector. 

### 2.2. Analysis of Crystallization

The thermophysical characteristics of the samples were determined by the DSC method using a differential scanning calorimeter DSC 204 F1 (Netzsch, Selb, Germany) under a nitrogen atmosphere. Samples of about 5.0–5.4 mg sealed in aluminum pans were heated from 20 to 200 °C at a rate of 10 °C/min. Calibration was carried out with Indium (T_m_ = 156.6 °C). The crystallinity degree of PLA (χ_c_) was calculated using the following equation:

χ_c_ (%) = (Δ*H*_m_/Δ*H*_m_*) × 100%

where Δ*H*_m_ is the melting enthalpy (an experiment result) and Δ*H*_m_* is the enthalpy assuming 100% crystalline PLA homopolymer 93.1 J/g [28].

### 2.3. Electronic Paramagnetic Resonance

EPR spectra were recorded on an automated EPR-V spectrometer (Semenov Federal Research Center for Chemical Physics, RAS, Moscow, Russia). For in order to avoid saturation effects, microwave power in the resonator was maintained below 7 mW. When recording spectra, the modulation amplitude was significantly below the resonance line width and did not exceed 0.5 Gs. The probe was a stable nitroxide TEMPO radical. The radical was introduced into the fiber from vapors at a temperature of 70 °C. The concentration of radicals in the polymer did not exceed 10^–3^ mol/L.

The value of τ_c_, which is the probe rotation correlation time, was calculated according to the equation [29]:

τ_c_ = ∆H_+_ × [(I_+_/I_−_)^0.5^ − 1] × 6.65 × 10^−10^,

where ΔH+ is the width of the low-field component of the spectrum and I_+_/I_−_ is the intensity ratio of high- to low-field components, respectively.

### 2.4. Morphology of the Sample

The morphology of the initial PLA and PLA/NR fibers and after degradation was characterized by scanning electron microscopy Philips SEM-500 (Eindhoven, The Netherlands) at different magnifications. The surface and lateral sections of the samples were examined.

### 2.5. FTIR-ATR Spectroscopy

The FTIR spectra of investigated samples were recorded on a Bruker Lumos IR Fourier microscope (Bruker Corp., Bremen, Germany) at a room temperature of (22 ± 2) °C in the range of wave numbers 400–4000 cm^−1^. The description of the band intensities is given in the text. The analysis was carried out by attenuated total reflection (ATR) using a diamond crystal.

### 2.6. Thermogravimetric Analysis

TGA-DTG measurements were performed under an argon environment using a thermogravimetric analyzer TG 209 F1 (Netzsch, Germany). All fiber samples were heated from 50 to 800 °C with a heating rate of 20 °C/min.

### 2.7. Soil Test

In the same containers, 200 ± 20 g of the soil of the brand “Soil Keva Bioterra” (Gera LLC, Lytcarino, Russia) with pH 5.5–7.0 were used. Content of active substances was (N) ≥ 275 mg/L, (P_2_O_5_) ≥ 325 mg/L and (K_2_O) ≥ 325 mg/L. The experiment was carried out at a temperature of 25 ± 2 °C. During the experiment, soil moisture was maintained at 55–60%.

### 2.8. Water Uptake

The kinetics of absorption of distilled water by PLA/NR samples was studied for 288 h until the materials reached equilibrium water absorption (ISO 62: 2008). For testing, square-shaped film samples with a side of 30 mm were used. The test was carried out on at least three samples of each composition. Before testing, the samples were dried at 40 ± 2 °C for 24 h, and then cooled in a desiccator over a desiccant—calcium chloride at 22 ± 2 °C and weighed no more than 5 min after removal from the desiccator. Next, the samples were placed in a vessel with distilled water, taken in an amount of at least 8 cm^3^ per 1 cm^2^ of the sample surface. The test samples did not come into contact with each other, as well as with the walls of the vessel, and were completely covered with water. The liquid was stirred by rotating the vessel at least once a day.

After reaching an equilibrium amount of water in the sample, it was removed from the water, dried with filter paper, and weighed on a balance (Acculab Atl-220d4-I, AG, Germany) no more than 1 min later. The equilibrium degree of water absorption was considered achieved if the change in mass values was within 0.1%.

The degree of water absorption was calculated as:

$$W=\frac{\left(m_{2}-m_{1}\right)}{m_{1}}\times 100\%$$

where *m*_1_ is the initial mass of the sample and *m*_2_ is the mass of the sample after water influence.

### 2.9. Statistical Processing

Experimental data was calculated as the arithmetic mean and its standard error. The calculations were executed using Statistica 8.0 software (Dell Software Inc., Round Rock, TX, USA) and Microsoft Excel 2007.

## 3. Results

### 3.1. Morphology

**Figure 1:**
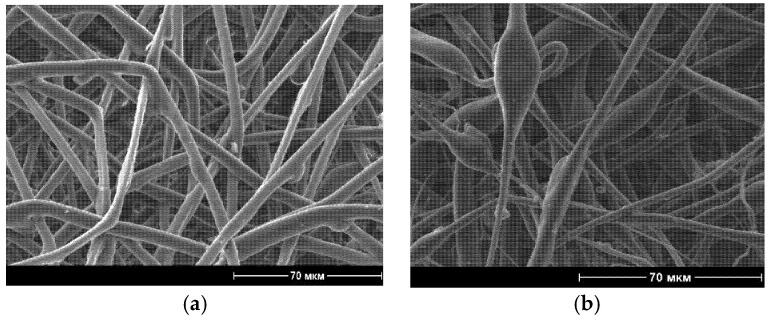

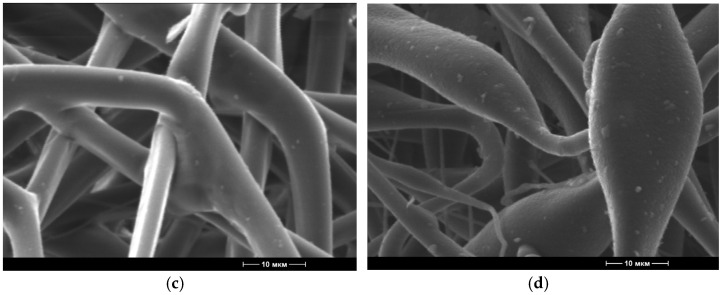
Scanning electron micrographs of PLA (**a**,**c**) PLA/NR fibers with 15 wt.% (**b**,**d**) of NR content at different magnification.

When NR was added to the PLA matrix, the morphology of the non-woven fibers changes. This fact is studied and discussed in papers [15,28,30]. For example, Figure 1 show non-woven materials made of pure PLA and PLA/NR with an NR content of 15 wt.%.

The appearance of all samples did not vary, but SEM micrographs show diversity in the fiber morphology. Due to the difference in viscosity of PLA and NR, the rapid evaporation of the solvent and the nonequilibrium state of the polymer system, a bead morphology was observed during electrospinning [15].

The resulting electrospun fibers were used as a substrate for growing basil (*Purple Ruffles*) in the phytotron. After the basil vegetation process (60 days), the plants were carefully removed, and part of the polymer substrate samples with different NR content were taken to conduct experiments on changing the structure during degradation in the soil. The rest of the samples of PLA/NR fibers were kept in the soil for another 160 days and then also examined. Isolation of soil micromycetes was carried out by the method of serial dilutions on solid nutrient media. Micromycetes of the *Aspergillus* spp., *Trichoderma* spp., *Penicillium* spp. were isolated. These molds are very aggressive to polymer materials. *Trichoderma* spp. is a fungal PLA degrader; it is classified as white rot micromycete, being present in all soil [31]. Mold contamination by the *Aspergillus brasiliensis* and *Penicillium* *chrysogenum* of fibrous and film samples of PLA was also active [22].

**Figure 2:**
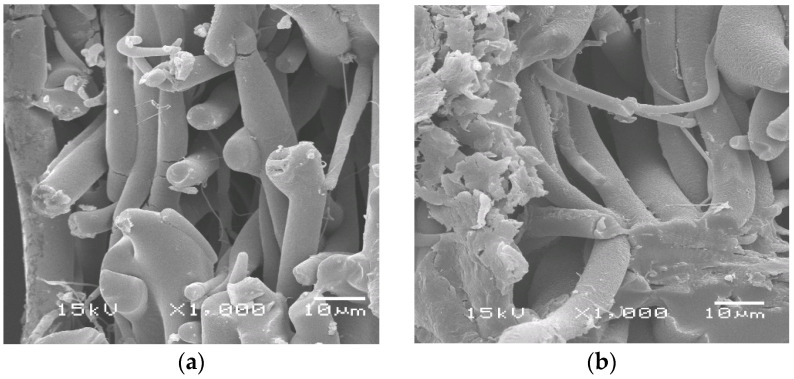

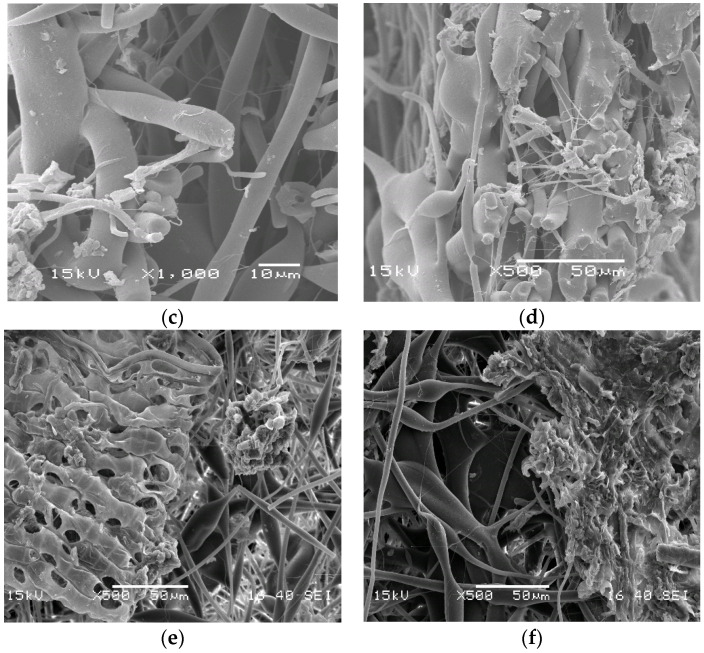
Scanning electron micrographs (a lateral view) after 220 days of soil exposure of PLA/NR samples with different NR content, wt.%: 0 (**a**), 5 (**b**), 10 (**c**,**e**), 15 (**d**,**f**). Micrographs (**e**,**f**): a surface view.

The microphotographs (Figure 2) show a lateral section of the investigated fibers. After degradation in the soil, microcracks and roughness are noticeable, but serious damage by microorganisms is difficult to observe. That is because the deeply destroyed material crumbles at a lateral cut. Nevertheless, in the photos of the surface, one can see significant damage (Figure 2e,f). (See also the Supplementary Materials). There are also changes in the spectral and thermal characteristics of the fibers.

### 3.2. Crystallization Analysis

**Figure 3:**
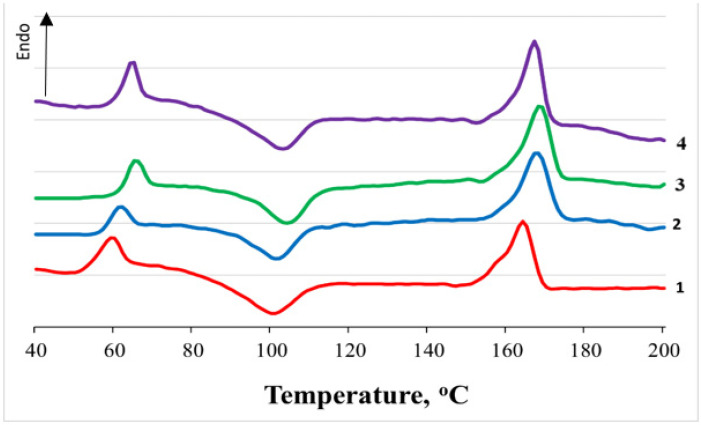
DSC thermograms of initial PLA/NR samples with different NR content, wt.%: 0 (1), 5 (2), 10 (3), 15 (4).

The thermophysical characteristics of non-woven PLA and PLA/NR fibers were determined by the DSC method before and after degradation in the soil for 60 and 220 days (Figure 3 and Table 1). Features of the PLA/NR fibers’ structure were discussed in detail in [15].

In the process of degradation in the soil, a certain pattern of changes in the thermophysical values of pure PLA and samples of non-woven PLA/NR fiber was noticeable. The values of PLA melting (T_m_), glass transition (T_g_) and cold crystallization (T_cc_) temperatures increased, and these temperatures also decreased in composites. This was especially noticeable in samples with an NR content of 10 and 15 wt.%.

An interesting result was observed regarding the crystallization temperature. In the initial fiber samples, the T_cc_ increased with rising the content of natural rubber, but after exposure to the soil, the opposite dependence can be observed. Apparently, in the process of biodegradation, defects accumulate in the main chains of the polylactide, and the degree of supercooling of the system for the formation of crystallites should be higher.

An increase in the PLA degree of crystallinity is a consequence of the disintegration of the amorphous phase and a possible decrease in the molecular weight. When NR is added to the PLA matrix of the initial samples, the PLA degree of crystallinity PLA increases [30,32]. When exposed to the soil, the PLA degree of crystallinity in PLA/NR fibers decreases, which indicates the appearance of defective crystal structures during degradation.

The role of water cannot be ignored: water is directly involved in the degradation process in the soil. The combined effect of water and soil microorganisms leads to a change in the morphology and structure of PLA/NR fibers. One of the characteristics influencing the process of biodegradation is the degree of water absorption.

**Figure 4:**
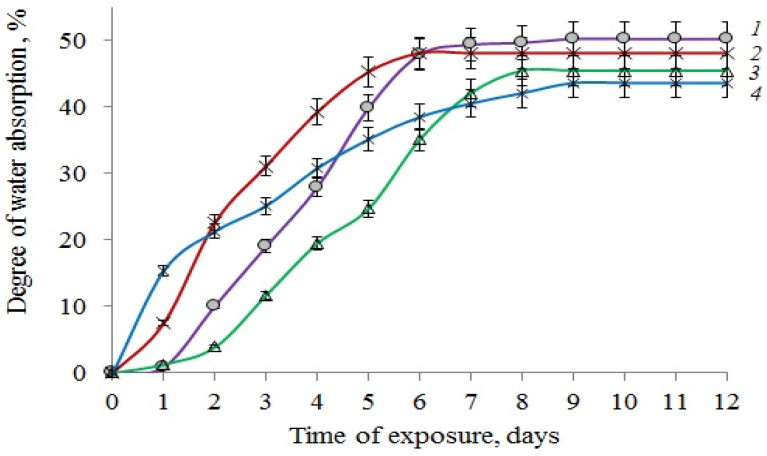
Water uptake of PLA/NR non-woven fibers. The content of NR, wt%: (1) 0; (2) 5; (3) 10; (4) 15.

Figure 4 show the water absorption kinetics of PLA/NR fibrous materials. The addition of NR reduces the degree of water absorption of PLA/NR samples compared to 100% PLA by several percent. Possibly, the beaded morphology formed in the PLA/NR fibers somewhat hinders the absorption of water. It should be noted that the effect of water on the thermophysical characteristics of PLA/NR fibers outside the soil is more active. So, when exposed to water for 180 days at T = 22 °C, the DSC thermograms of the studied samples did not contain the peak of glass transition and cold crystallization of PLA [30]. In the experiment with soil, no such effect was observed, although water was also involved in the biodegradation process.

### 3.3. Macromolecules’ Dynamic

**Figure 5:**
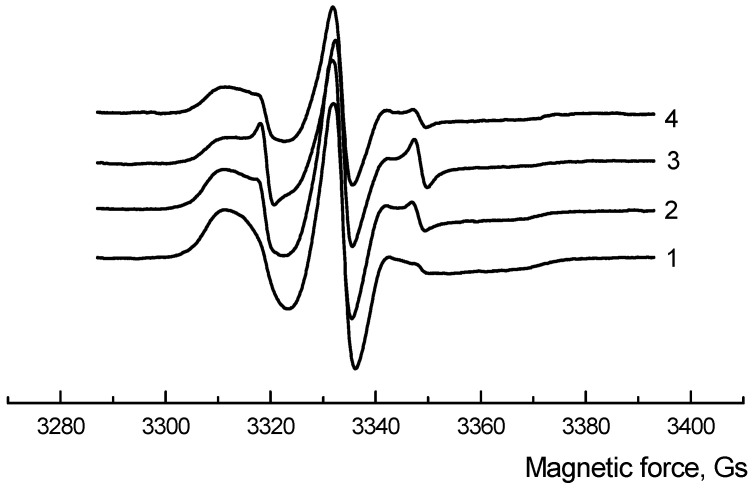
EPR spectra of initial PLA/NR non-woven fibers. The content of NR, wt.%: (1) 0; (2) 5; (3) 10; (4) 15.

The changes in the macromolecular mobility of the amorphous phase in the PLA/NR fiber samples were controlled by the EPR method (Figure 5). The radical was injected above the glass transmission temperature of the PLA at T = 70 °C to avoid the glassy state of the polymer.

The time of the probe-radical correlation for the initial PLA/NR samples and after degradation in the soil for 60 and 220 days are shown in Table 2.

**Figure 6:**
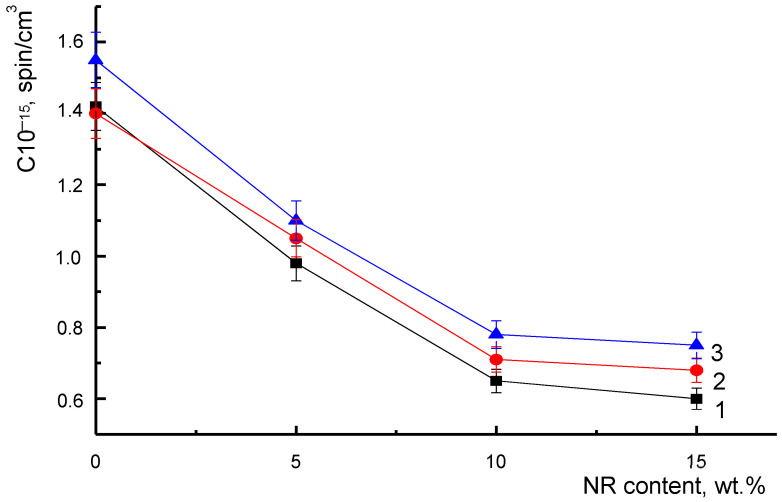
Radical concentration in PLA/NR fibers of initial (1) and after 60 days (2) and 220 (3) of soil exposure.

In the initial samples, the correlation time in the compositions was reduced by three–four times as compared to the PLA. The results obtained by the EPR method were in agreement with the DSC data: the degree of PLA crystallinity increased in the presence of NR due to a rise in segmental mobility [32]. However, the increase in the degree of crystallinity was not as significant as the change in the correlation time in the samples. After degradation in the soil for 60 and 220 days, the values of the PLA correlation time decreased, and those of the PLA/NR fibers increased. To explain this fact, the concentration of the radical adsorbed by samples of the same mass was determined (Figure 6).

The dependence had a non-linear character. The concentration of the radical in the PLA fibers was the highest, and with the addition of 5 wt.% of natural rubber in the composition, it decreased sharply. With a natural rubber content of 10 and 15 wt.%, the change in the radical concentration was smoothed out. A different trend persisted after the degradation of the studied samples of non-woven fiber in the soil. During the degradation process, under the influence of moisture and enzymes secreted by basil roots, the ratio of amorphous and crystalline phases changed with the alteration of the amorphous phase itself. Thus, with an increase in the decomposition time of the samples in the soil, the radical penetrated deeper into the amorphous areas of the fibers at a higher concentration.

**Figure 7:**
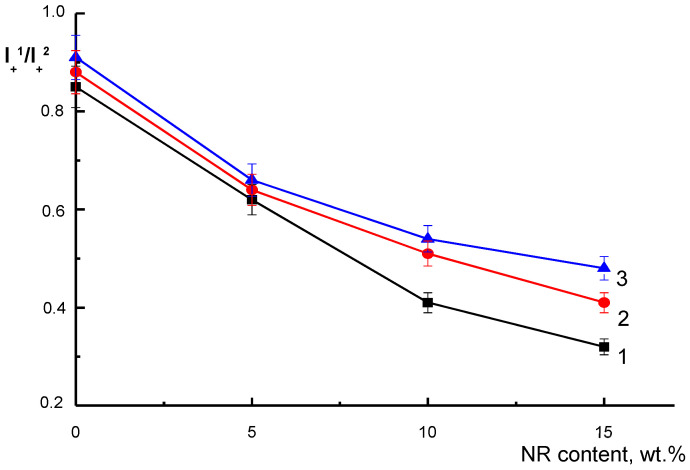
I_+_^1^/I_−_^2^ value in PLA/NR fibers of initial (1) and after 60 days (2) and 220 (3) of soil exposure.

Changes in the structure of the polymer matrix are reflected in the I_+_^1^/I_+_^2^ value, which characterizes the ratio of the slow and fast constituents of the amplitude high-field component (I_+_) (Figure 7).

In the original samples, with an increase in the NR content, the I_+_/I_−_ ratio decreased, i.e., the proportion of dense regions reduced, and the fast component of the spectrum increased. The effect of the soil did not change the type of concentration dependence. However, the I_+_/I_−_ values increased for each sample over time. Consequently, after 60 and 220 days of exposure, the least ordered regions were destroyed, and the radical penetrated deeper and deeper into the dense intercrystalline regions.

### 3.4. Thermal Degradation

**Figure 8:**
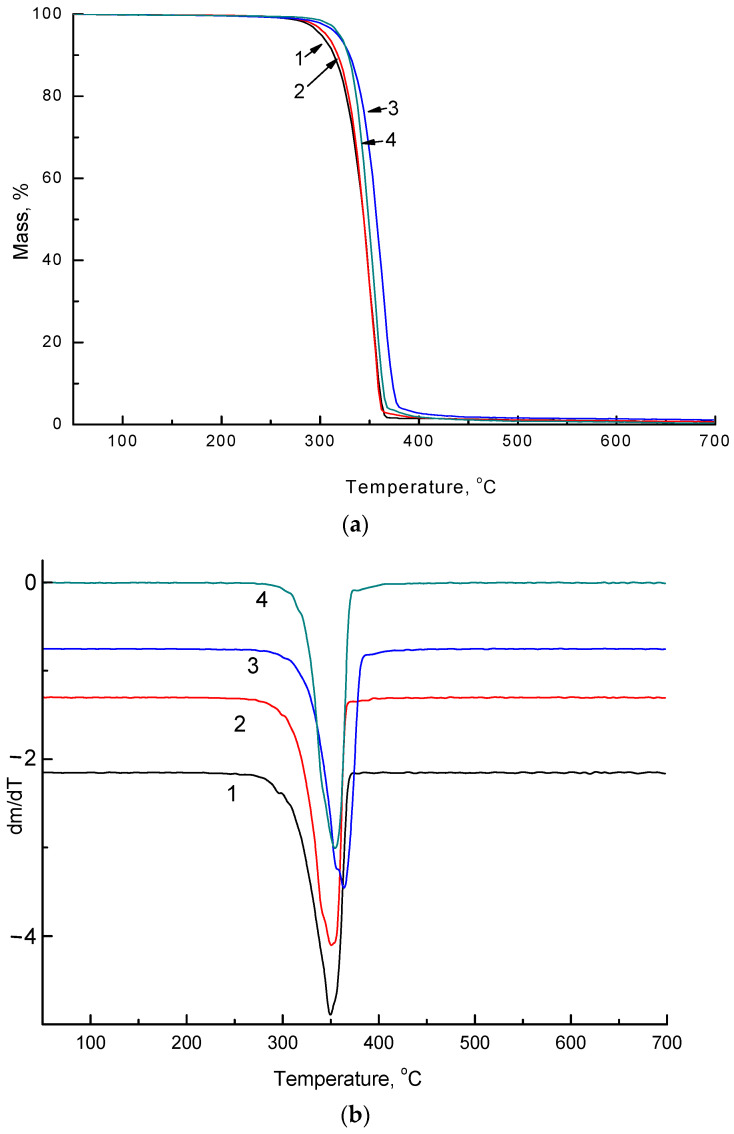
TGA (**a**) and DTG (**b**) thermograms of PLA/NR samples with different NR content, wt.%: 0 (1), 5 (2), 10 (3), 15 (4).

**Figure 9:**
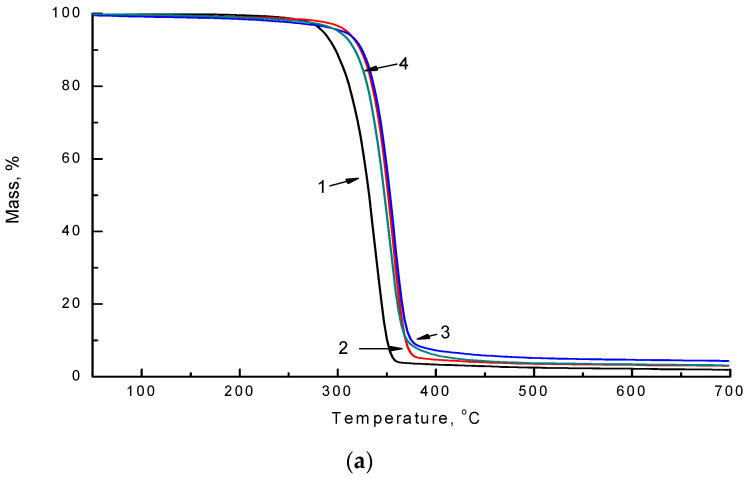

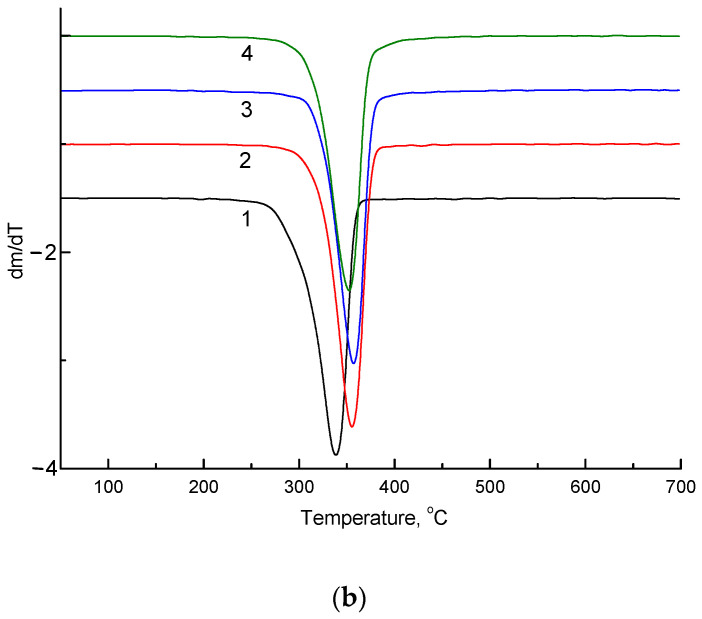
TGA (**a**) and DTG (**b**) thermograms after degradation in the soil during 220 days of PLA/NR samples with different NR content wt.%: 0 (1), 5 (2), 10 (3), 15 (4).

The impact of the soil is reflected in the thermal properties of non-woven PLA/NR fibers. TGA thermograms of the initial samples and after degradation in the soil are represented in Figure 8 and Figure 9.

The process of thermal degradation may be divided into three stages. The first stage is a small level of weight loss, which may be associated with the loss of moisture in the samples. The second stage is the main decomposition stage (300–390 °C), where most organic materials decompose, and maximum weight loss occurs. The third stage is the carbonization stage, with a slight weight loss, in which some carbonaceous fractions undergo further decomposition [33]. The values of the thermal degradation characteristics obtained by TGA are listed in Table 3.

According to the experiment, the onset temperature of thermal degradation increases with the addition of NR to the PLA matrix since natural rubber is more thermally stable than pure PLA. Natural rubber starts to degrade at a temperature of 350 °C. After using non-woven fibrous materials as a substrate and exposure to the soil for 220 days, the onset temperature values changed significantly. For all samples, the onset temperature shifted to the region of lower temperatures. The most significant decrease in the starting temperature of the decomposition process was observed for a sample with an NR content of 15 wt.%. The T_max_ (maximum weight loss temperature) decreased by 11 °C in the PLA sample. In the PLA/NR composites, the values of T_max_ decreased by approximately 4 °C. It is shown that the DTG curves of PLA/NR fibers (Figure 8b and Figure 9b) have one peak, denoting one decomposition process. Apparently, the NR content is not large enough to see the individual peak of its decay.

### 3.5. FTIR Spectroscopy

**Figure 10:**
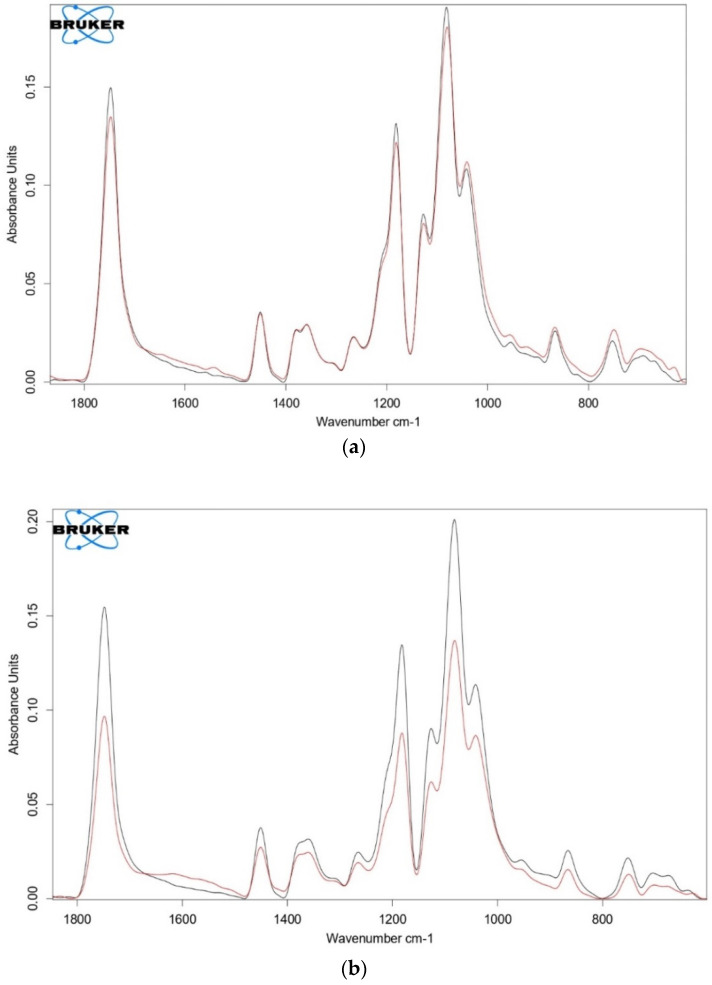
FTIR spectra of PLA (**a**) and PLA/NR fiber (**b**) with the NR content 10 wt.%: grey—before, red—after degradation in soil for 220 days.

Control of the structure of PLA and PLA/NR fibers was also carried out by FTIR spectroscopy. For example, the spectra of 100% PLA and PLA/NR fibers with an NR content of 10 wt.% are presented (Figure 10).

In all PLA/NR samples, after exposure to the soil, the intensity of structurally sensitive bands decreased (Figure 10b). Absorption bands 1380–1000 cm^−1^ belong to spatial fluctuations –C–O-groups. In the interval 1900–1600 cm^−1^, a peak of 1745 cm^−1^ is allocated, which belongs to the –C=O groups [34]. The decrease in the intensity of these bands, apparently, occurs due to the elimination of ether groups in the process of degradation. Bands 755 and 860 cm^−1^ corresponding to the crystalline and amorphous phase of PLA are also less intense after degradation in the soil [35]. In 100% of the PLA, the intensity of the above bands increased slightly, and the completion of crystal formations occurred. These data confirm the results of DSC (Table 1): the degree of crystallinity of PLA fibers increases after degradation in the soil. Attention should be paid to the area of 1660–1550 cm^−1^, which refers to the deformation vibrations of N-H and C-N groups in primary amides and the structure of proteins (Figure 10b). The intensity of the bands in this region increased due to the splitting of the rubber macromolecules by bacteria [36,37].

## 4. Discussion

The interfacial interaction in the polylactide-natural rubber system was discussed in [15]. The biotic degradation of PLA/NR fibers is undoubtedly influenced by the ratio of components and the structural features of the resulting composites. Based on the degradation process, the incompatibility of the polylactide-natural rubber system is an advantage. Heterogeneous structures are usually destroyed faster than homogeneous ones. Boundary layers and near-boundary areas are characterized by lower density and greater accessibility for aggressive agents. For instance, Arrieta et al. studied the degradation in the compost of plasticized PLA and its composites with PHB. Mixtures of PLA and PHB are thermodynamically incompatible. In that work, after 21 days of biodegradation, more active destruction of samples of PLA/PHB blends was observed compared to PLA [38]. In the presence of an incompatible component, the integrity of the polylactide matrix is disrupted, and such a heterogeneous heterophase system is destroyed more rapidly.

It is obvious that NR influences the process of PLA degradation. Melting and glass transition temperatures in samples of fibrous PLA/NR of all compositions tend to decrease, and in 100% of PLA, increase. Usually, during the disintegration of the polymer, the value of the glass transition decreases. The authors of [39] report that the absorbed moisture during weathering induces the plasticization of the polymer, which increases the mobility of the chains resulting in lower glass transition temperatures. Probably, in this experiment, the degree of degradation of PLA was not deep enough, and a process similar to polymer annealing is observed, in which crystallizing polymers have an increase in the degree of crystallinity and glass transition temperature. Moreover, the crystalline grains have a high packing density of macromolecules and are resistant to aggressive environmental factors. Their degradation is associated with low permeability for all types of low molecular weight liquids and gases [40].

The dynamics of macromolecules is also changing. In the process of degradation in the soil, moisture and microorganisms first attack the amorphous phase of fibrous composites. Any polymer and polymer composites are characterized by the presence of areas with reduced density. In crystallizing polymers, such a region is the amorphous phase, which acts as a trap for radicals and the progenitors of submicroracks. An increase in the correlation time is associated with the degradation of amorphous regions and the penetration of the probe radical into denser interfibrillar regions. In the case of 100% PLA, a different effect is observed. The correlation time decreases after 60 and 220 days of biodegradation in the soil, which is due to changes in the volume and density of the amorphous phase of polylactide. It is possible that there is some increase in the mobility of macromolecular segments due to partial degradation of the amorphous phase and rupture of through chains. The difference in EPR results between PLA and fibrous substrates of PLA/NR proves the effect of NR on the structure and dynamics of the PLA matrix.

Due to the disintegration of NR and PLA, the onset temperatures of thermal degradation are shifted to the region of lower temperatures (TGA data). However, the patterns of thermal decomposition and the form of the TG curves of PLA/NR fibers do not change.

Degradation in the soil is the action of water, temperature, bacteria and molds at the same time. It should also be noted that the rapid acceleration of the degradation of such materials in the soil can be achieved by increasing the possibility of surface penetration of enzymes, i.e., by increasing the specific surface area, and especially wettability [41,42]. NR is unlikely to increase wettability, but it does increase the proportion of the amorphous phase in the PLA/NR fiber composites, increasing the vulnerability of the polymer matrix to the effects of aggressive environments. PLA easily hydrolytically decomposes with a change in structure and properties in various liquid media [40,43,44,45]. A temperature of 30–50 °C accelerates the degradation process [40]. In [46], in the process of studying the incubation of PLA in the soil under normal conditions, it was found that it slowly undergoes destruction, and the onset of decomposition occurs after several months. Works [47,48] show that there are significantly fewer PLA-degrading microorganisms in the environment than microorganisms that are capable of degrading poly-3-hydroxybutyrate and poly(ε-caprolactone). Other studies also demonstrated minimal PLA degradation at ambient temperatures. PLA is a compostable plastic. PLA can be degraded in a composting environment after 45–60 days at 50–60 °C by microorganisms in the compost [49].

The natural rubber is an elastomer of natural origin, and it undergoes biodegradation by various microorganisms. There are works aimed at studying the biochemical mechanism of the biological decomposition of natural rubber [50,51,52].

In Section 3.5, the band 860 cm^−1^ was discussed. It should be noted that this band overlaps with the 863 cm^−1^ band, which refers to the C-H- out-of-plane deformation oscillation of group C(CH_3_)=CH in the macromolecule of rubber. Reduction of the intensity of this band in the spectra of PLA/NR fibers confirms the degradation of both the PLA matrix and natural rubber. During the growing season, the basil root system releases enzymes that also affect the polymer substrate and change its structure and properties. However, this is the subject of further research.

## 5. Conclusions

Biobased electrospun PLA/NR fiber with an NR content of 5–15 wt.% was used as a substrate for the basil growing. Erosion of the surface of the samples was recorded by SEM. The main objective of this work was to study the thermal and dynamic properties of fibrous substrates before and after degradation in the soil at 60 and 220 days. The effect of the soil on the morphology of PLA/NR fibers is characterized by the formation of cracks and surface roughness. The DSC results indicate the changes in T_g_, T_m_, T_cc_ values. After 220 days of the soil exposure, the degree of crystallinity in pure PLA increases, while in PLA/NR fibers it slightly decreases. The TGA method showed a shift in the initial and maximum degradation temperatures towards low temperatures. The EPR method recorded a change in structural and dynamic characteristics. As the amorphous phase is destroyed, an increase in the probe-radical correlation time is shown because it penetrates into denser regions at a higher concentration.

## Figures and Tables

**Table 1:** Thermophysical characteristics of PLA/NR non-woven fiber samples: before and after 60 and 220 days of soil exposure.

NR, wt.%	T_g_, °C (Δ ± 0.5 °C)			T_m_, °C (Δ ± 0.5 °C)			T_cc_, °C (Δ ± 0.5 °C)			χ_cr_, % (Δ ± 1%)		
	0 d	60 d	220 d	0 d	60 d	220 d	0 d	60 d	220 d	0 d	60 d	220 d
0	61	62	62	164	166	166	101	104	106	33	38	37
5	63	63	64	168	167	166	102	104	103	36	35	35
10	66	61	62	167	167	165	105	102	100	37	36	35
15	65	62	62	166	165	164	104	100	99	36	34	33

**Table 2:** The correlation time of the probe in PLA/NR fibers. The radical was inserted at T = 70 °C.

NR, wt.%	The Correlation Time		
	τ_c_ × 10^−10^ c^−1^, initial	τ_c_ × 10^−10^ c^−1^, 60 days	τ_c_ × 10^−10^ c^−1^, 220 days
0	72.1 ± 0.19	55.2 ± 0.14	39.0 ± 0.23
5	28.2 ± 0.15	30.7 ± 0.20	38.7 ± 0.18
10	19.6 ± 0.12	27.8 ± 0.18	31.8 ± 0.12
15	17.4 ± 0.18	25.0 ± 0.15	31.0 ± 0.13

**Table 3:** Thermal degradation of PLA and PLA/NR samples: before and after 220 days of soil exposure.

NR, wt.%	Onset Temperature, °C (Δ ± 0.5 °C)		T_max_, °C (Δ ± 0.5 °C)	
	initial	220 days	initial	220 days
0	326.3	310.9	350.1	338.9
5	330.0	319.1	350.7	355.7
10	339.0	325.7	361.5	357.0
15	332.4	307.3	357.3	353.2

## Data Availability

The data presented in this study are available on request from the corresponding author.

## Supplementary Materials

The following supporting information can be downloaded at: https://www.mdpi.com/article/10.3390/polym14051058/s1, Figure S1. Micrographs of PLA/NR sample with 10 wt.% of NR content after 60 days of degradation in soil: (a) general view; (b) individual fiber view.
