# Supplementary material for: Thermal Properties and Dynamic Characteristics of Electrospun Polylactide/Natural Rubber Fibers during Disintegration in Soil
Source: Polymers (Basel). 2022 Mar 7;14(5):1058. doi: 10.3390/polym14051058 (PMC8914975; doi:10.3390/polym14051058)
Supplement: Supplementary file 1 [file polymers-14-01058-s001.zip › polymers-1549748-SI.pdf]

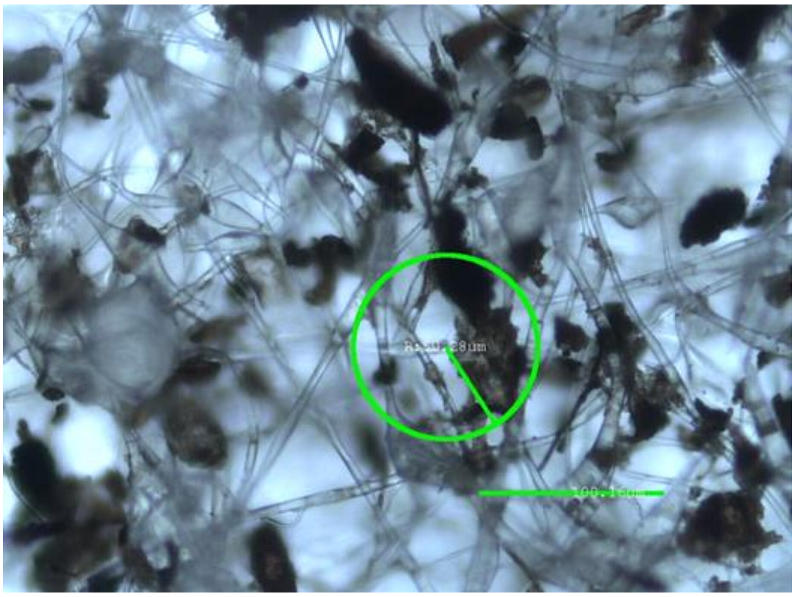

a

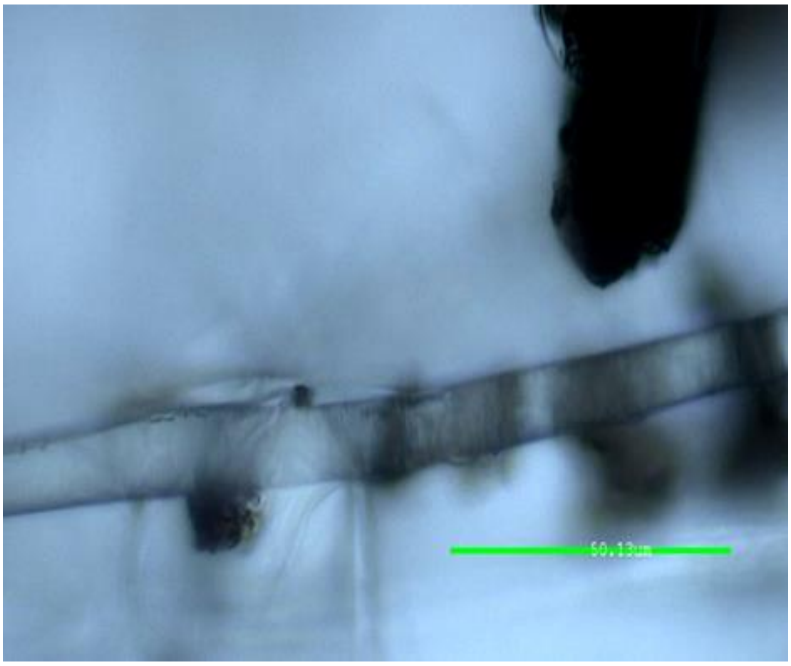

b

Figure S1. Micrographs of PLA/NR sample with 10 wt.% of NR content after 60 days of degradation in soil: (a) general view; (b) individual fiber view.

The samples were thoroughly washed. Darkening in the samples is a consequence of contamination by molds and bacteria.
